# Active aging and urban policies: the space as an instrument for an inclusive and sustainable city

**DOI:** 10.3389/fsoc.2023.1257926

**Published:** 2023-12-11

**Authors:** Letizia Carrera

**Affiliations:** University of Bari Aldo Moro, Bari, Italy

**Keywords:** aging process, urban policies, space, third space, social representations

## Abstract

Aging is one of the most important challenges of our times. As stated by United Nations’ report on 1983, “Policies to meet the challenge of a growing, healthier and more active seniors population -based on the view of the ageing of society as an opportunity to be utilized -automatically benefit the individual ageing person, materially and otherwise. Similarly, any effort to ameliorate the quality of life for the seniors, and to meet their diverse social and cultural needs, enhances their capacity to continue interacting with society”. Aging society provides not only a new context, but a new opportunity to rethink our traditional views of age. The growing number of seniors people will soon make the majority of overall population. As noticed by reports of international organizations, cities will play a more important role in dealing with these quantitative and cultural changes, mostly because it is expected that a quarter of the population over 60 will be concentrating in the central areas of compact cities. In brief, cities are at once growing and aging at an incredible speed. Although aging process represents a fundamental and structural phenomenon with very deep consequences at economic, social and political level, and with an impact on the individual one as on the society as whole, our cities should deal with this process and respond, in terms of public health and social care, to needs of older people, also those that will experience a loss of autonomy. It is important to note that there are a greater heterogeneity within older population in terms of conditions and demands, which depend on their specific personal, social and familial context. Urban space - in its complex differentiation between public space, third space, and private space - represents both a tool and a strategic factor in pursuing the objective of ensuring high levels of widespread well-being and, from a political perspective, fully shaping the right to the city for seniors individuals.

## Introduction

Aging is one of the most important challenges of our times (Attias Donfut et al., 2005; Moulaert and Paris, 2013). “Policies to meet the challenge of a growing, healthier and more active seniors population – based on the view of the ageing of society as an opportunity to be utilized – automatically benefit the individual ageing person, materially and otherwise. Similarly, any effort to ameliorate the quality of life for the seniors, and to meet their diverse social and cultural needs, enhances their capacity to continue interacting with society” (United Nation, 1983, p. 16). Aging society provides not only a new context, but a new opportunity to rethink our traditional views of age. Even going beyond these. As clear from many decades “our society is changing in many ways that relate to age. Perceptions of the periods of life are being altered, as well as role transitions, social competencies, and the ages that mark their boundaries” (Neugarten and Neugarten, 1986, p. 32). The growing number of seniors people will soon make the majority of overall population. As noticed by reports of international organizations (WHO, 2007; OECD, 2015, UN AGENDA 2030), cities will play a more important role in dealing with these quantitative and cultural changes, mostly because it is expected that a quarter of the population over60 will be concentrating in the central areas of compact cities. In brief, cities are at once growing and aging at an incredible speed. Although aging process represents a fundamental and structural phenomenon with very deep consequences at economic, social and political level, and with an impact on the individual one as on the society as whole, our cities seem not to be ready to deal with this process and to prevent possible issues.

Progressive increment in share of older adults population increasingly raises the question of how governments will respond, in terms of public health and social care (Galasso, 2006; Bruggencate et al., 2018), to needs of older people, and especially to those that will experience a loss of autonomy. It is important to note that there are a greater heterogeneity within older population in terms of conditions and demands, which depend on their specific personal, social and familial context (Neugarten and Neugarten, 1986; Pavolini, 2001, 2004; Ranci, 2001; Ranci et al., 2019). These differences and multiple images of age groups make every label and every bounder very fluid. “Older persons are very heterogeneous in social, political and economic terms” (Neugarten and Neugarten, 1986, p. 42). Understanding needs of older citizens should not be limited, however, to health plans. Today more than ever before the prevention and care plans must include a positive impact on the quality of life for these people starting from the need of a cultural change from “cure” to “prevention” of social isolation across the life-course (Cotterel and Buffel, 2018, p. 1). In this perspective cities and their choices about urban age policies and urban habitat plays a very important role.^1^

The role of urban welfare is, therefore, increasingly crucial to address and respond to such needs, as well as to the new and more complex demands of fully independent older people. These entail a range of requests, from mobility, care, safety, and proximity, to an environment tailored to their changing needs and an equipped public space, but also urban green spaces, leisure and sociality opportunities, what can be summarized with Henri Lefebvre’s notion of an all-encompassing “right to the city” (Lefebvre, 1967; Harvey, 2016). The unprecedented centrality of older people in urban space helps us to conceive of this right to the city as something more than a differentiated and personalized use of the city itself giving them a full centrality in defining policies and socio-urban interventions.

The recent Agenda 2030 of the United Nations has fully acknowledged this new political and social centrality of older people among its objectives, which include a focus on their right to an inclusive and accessible city and recognize urban spaces as physical structures that can deeply affect their daily life. It’s a focus goal to make social and/or physical environments more conducive to older adults’ health, wellbeing, and ability to age within their places and their communities (Greenfield et al., 2019). To use an expression of Nigel Thrift (2016), the built space is “actively passive,” because it is made and, simultaneously, capable of making, in other words, it is a structured and structuring entity. Once built, our cities continue to influence social relations, different levels of power and inequality. In this perspective, city, considered as meso level, can influence the highest macro level of society (Greenfield et al., 2019). As Giandomenico Amendola points out, the shape of the city has an impact on the structural conditions of everyday experience:

“As a structuring device, the built space organizes and defines practices, thus contributing to the reproduction of social relationships. Both the purely physical dimension of spatial organization and the symbolic one, therefore, contribute not only to “suggest” certain uses of space and specific social relationships, but also to give concrete form to the life that takes place there. (...) The physical environment is linked to the knowledge and the dominant powers in the society by a mutual connection” (Amendola, 2009, pp. 39, 43).

The demand for a high-quality urban space is a key point in the process of re-signification of status of third and fourth ages. For their strategic value in relation to issues of safety and accessibility for seniors people, city should increasingly be in the focus of public policies (Zajczyk, 2000, 2018) without thinking yet at “societal resources invested in betterment of the ageing as a drain on the economy more than a productive investment having both tangible and intangible benefits” (D’Souza, 1993, p. 342). Also, organization of public space and suitability and availability of infrastructures for mobility across the city are crucial in defining quality of life, social relations and sense of community for different types of citizens, especially for those more socially fragile and vulnerable, like older adults (Esping-Andersen, 2000; Zajczyk, 2000, 2018; Pavolini, 2001; Emerijck, 2002; Bonoli, 2004).

In view of aging process that our societies currently face, and will be facing more pressingly in the coming years, cities and urban policies play a decisive role in achieving the conditions for sustainable, inclusive and age-friendly city, pursuing the guiding principle that cities must become more and more friendly for all ages (WHO, 2007; Whal and Oswald, 2010; Settersen and Angel, 2011; Handler, 2015).

## Quantitative and qualitative changes in the aging process: the *new seniors*

In line with a European and world trend, Italy is becoming an increasingly old country. At the beginning of 2022, seniors people over 65 years-old were more than 14 million, which amounts to 3 million more than 20 years ago. Current forecasts for the 2042 confirm this trend, as this number is expected to grow up to 19 million by then. Besides, the aging trend appears to be growing strongly in the demographic projections that indicate an average of 41.9 years in 2002, 45.7 in 2020, at least 47.2 years in 2030, 48.2 in 2040 and even 48.6 in 2050 (Istat, 2020). As in the other European countries, the curve of incremental aging of the Italian population is influenced by both the declining birth rate and the rising life expectancy. According to Istat projections, the share of over-65 s in Italy could increase of 9 or 14 percentage points in 2050, depending on more or less optimistic assumptions. However, these figures do not differ significantly from the European averages reported by Eurostat, which shows evidence of a widespread and structural process that needs to be addressed. These important quantitative changes have to be combined with substantial qualitative ones in social representations of third age and, therefore, in meaning that being seniors comes to assume. “In recent decades, being seniors has begun to be considered no longer as a mere residual condition against the active role of adult workers, but rather a completely new phase full of opportunities and possibilities, but also of limits and difficulties” (Carrera, 2022b, p. 3). Lidia Ravera writes: “third half of life” is a good time to practice changing” (Ravera, 2023, p. 12), reflecting on the possibility, referring above all to women, of valuing third age as a moment of fullness and self-esteem. Urban policies and responses to life changes that they enable are responsible to determine the ways in which old age can be experienced. In absence of structured plans with clear objectives to secure the conditions for an active aging and a meaningful life for seniors people, quality of life is almost exclusively dependent on resources owned individually, in terms of economic, cultural and social capital. The risk is to deny some citizens, the poorest ones, right to a substantial equality of opportunities and *capabilities* (Sen, 1986, 1992; Nussbaum, 2002).

It is equally important to stress that a discussion of aging implies an awareness of the huge variety of meanings that can be found under the label of the “old age” in social, political and economic terms (Neugarten and Neugarten, 1986; Gilleard and Higgs, 2002). The reference to defined time intervals, in fact, shows how old age, or rather aging, should be considered more as a process. And it involves a wider notion of well-being, both experienced and perceived, and less as a circumstance linked to health in the strict sense (Caradec, 2001a,b; Bai, 2014; Carrera, 2020). The boundaries are becoming fluid and some age events are ceasing to represent a marker of social age (Moulaert and Biggs, 2012). There are the “young old” (65–74 years old), the “old” (75–84 cohort) and the “old old/seniors” including people over the age of 85, but also the “oldest old,” that is, over 90 years-olds, the over centenarians and the super-centenarians (one hundred and ten years-olds and over) (La Pierre and Keating, 2013; Greenfield et al., 2019). These categorizations show the need to recognize the great diversity of subjectivities among older people and their internal difference in terms of lifestyles, experiences, needs, desires, projects (Börsch-Supan and Jürges, 2005; Croda and Gonzáles-Chapela, 2006; Alwin and Hofer, 2011). Third age can be indeed characterized either by a feeling of fulfillment, new plans and a renewed search for meaning, or by fragmentation, loss of the self, loneliness and isolation, and even marginalization and social exclusion.

Socio-economic and cultural resources, including those offered by the urban space, can have a profound influence on the outcome of the gradual and inevitable deterioration of physical health. Individual and contextual factors are deeply interrelated and can heavily determine the quality of life of seniors people. Among the individual factors beyond age and more than this, there are very heavy factors such as the state of health, the level of autonomy, the endowment of economic and cultural capital, the quality of personal relationships with family and friends. The contextual factors, on the other hand, concern the provision of services across different urban areas in terms of quality and accessibility of public spaces, plans for public transport and mobility, safety and availability of green spaces and equipped parks, the perceived level of safety in the city, places for culture, leisure and socialization opportunities. We cannot forget the importance and the potentialities of technological innovations in order to create new relational spaces and occasions for the older people. “Such interventions have successfully alleviated social isolation by connecting older adult to their friends and the family online, whilst increasing younger adults’ self-esteem and confidence” (Cotterel and Buffel, 2018, p. 5). There is no doubt that advances in and increasing usage of social technology among older people highlight the potential of this medium for combating social isolation in later life. Equally important is the specific technology designed for the older people such as dedicated apps and home automation.

The experience of the third age, then, can be summarized as the complex outcome of the interrelation between these factors and the city features.

The aging process, thus, represents an additional and more complex level of challenge for urban policies because it puts to the test their effectiveness in securing opportunities and services, beyond the usual requests wishes of the citizens and perceiving a new and more complex goal of wellbeing connected to physical functioning (Moran et al., 2014); social and physical functioning (Bowling, 2007); social participation and volunteering (Dury et al., 2016) and mental health (Gale et al., 2011).

This generates an ongoing need for integrated territorial welfare services aiming at enabling the conditions for an active life, which means not just free from disease, but with plenty of opportunities available. Older people claim for needs that go far beyond the wish of keeping a good health. They look at the cities to fulfill their desires of sociality, cultural activities and leisure.

Urban welfare and good management of urban spaces are strategic aspects to secure an extensive right to the city for older adults, and anticipate new and unthinkable needs, its goal being a full implementation of their social and urban centrality.

## The role of urban spaces in designing an inclusive and age friendly city

The urban space and its organizational models are key factors to interpreting phenomena and changes and dealing with them (Gieryn, 2000) and to cope the challenge of ageing process related with urban space (Greenfield et al., 2019), time plans (Elder et al., 2003), and the consequences of regeneration of some specific urban environment as the process of gentrification (Lees et al., 2015).

In this perspective, urban spaces and its policies acquire a growing political relevance focusing on the aim of “describe, explain and modify/optimize the relationship between the ageing person and his/her physical environment” (Whal and Oswald, 2010, p. 112). They can be defined either in relation to a material morphology (the territory, in its practical-physical sense) and in relation to the symbolic value attached to the opportunity for practices and possibilities that they open up (Ikas and Wagner, 2008). A key turning point was the launch, in 2007, of an international network of ‘Age-friendly cities and communities’ (AFCC), a strategy of the World Health Organization, which is now a leading policy approach in many countries and cities (WHO, 2007, 2018; OECD, 2015). The attention due to the ageing process started to be related to the older people wellbeing more than to their capability to continue to work. Their active ageing ceased to be connected with job becoming a cultural, social and relational issues.

The awareness around the centrality of urban space in supporting a functional ageing process lies at the heart of the WHO’s choice to start the program of age-friendly cities with a twofold objective: firstly, to support the exchange of information between cities in order to speed up their transformation into suitable centers for seniors people, and secondly, to provide guidance for the development of related policies at regional and local level (Carrera, 2022a,b). The initiative began in 2006 with a preliminary analysis of various cities around the world and an evaluation of the facilities and services that make a city really age-friendly. The first step of the program was to set up a commission with representatives from 33 cities in 22 countries around the world, which produced a guide and a checklist, aimed at collecting senior citizens’ testimonies. For several years, the data so gathered highlighted the changes that would make cities more suitable for people over the age of 60 (Dehan, 1997; Bellanger, 2000; American Institute of Architects, 2004).

Within this strategy, the achievement of well-being involves an active role of public institutions at different territorial levels in securing the necessary material conditions of these new goals (Amendola, 2018). As noted, for older people as for any other groups of citizens, the variables affecting the quality of life are both personal and contextual.

Starting from recognizing the significant individual differences within the category of the older, is essential to ensure that the urban space can providing appropriate opportunities and a life of quality implementing differentiated urban welfare policies (Baltes and Mayer, 1999; Lalive d’Epinay et al., 2000; Caradec, 2001a,b; Gilleard and Higgs, 2002). The features of the spatial context – the *built environment* in particular – are extremely powerful factors that can either amplify or counteract *frailty* itself (e.g., allowing or preventing accidents at home or outside); they can either support or hinder *wellbeing* (e.g., feeling comfortable and safe at home or outside), they can increase or diminish material and social *isolation* (e.g., the feeling of loneliness and difficulty in going out and nurturing social relationships; Arlotti and Cerea, 2021).

The built environment and the residential context at large are fundamental conditions of the quality of daily life in view of the full right to an active aging and to aging in place (Golant, 2013). The former is important to protect the citizens, and among them the older ones, from the risk of social isolation and feeling of loneliness^2^ and help them to preserve their relationships with family, friends and neighbors.

The quality of the city is not the only key factor in shaping the citizens’ daily practices. The specific characteristics of the areas in which they live can also be decisive if there are problems of access to mobility. The demands of the older are particularly concerned with proximity, even when they live in an age-friendly urban habitat. To maintain a good level of life quality in the different neighborhoods of the city, it is essential to ensure the right to a high-quality aging process that implies the right to aging in place (Pani-Harreman et al., 2000; Moreno, 2020).

For older people, more than for other groups of citizens, proximity is a strategic aspect that includes both functional and relational features (Sennett, 1992a,b; Murray et al., 2010; Manzini, 2021). These are fundamental to ensure a urban habitat and networks of quality, but they should also be integrated with the model of diversified proximity “that offers many different opportunities and that allows to find (almost) everything a person needs to live” (Manzini, 2021, p. 15).

In order to achieve the goal of a full urban and social citizenship for older people, space must be considered and used as a strategic tool. If public space is a fundamental key to build a socio sustainable environment, private spaces also play an important part in this aim and should, therefore, be included in urban spatial policies.

### The public space

The quality of public space is a strategic tool to overcome the risks of isolation and the feeling of loneliness, mostly, older people face starting from the persistence of some traditional self-representations, the condition of loss of relationships linked with retirement, a reduced form of mobility, health conditions and autonomy that make higher the risk of isolation. Urban space should be accessible and adequately equipped with the necessary infrastructures and transport network made adequate also for the most vulnerable citizens. In other words, the aim of improving the quality of public space need to create and ensure concrete conditions to allow the older a meaningful use of urban spaces (WHO, 2018; Buffel et al., 2019; Del Barrio et al., 2021; Rémillard-Boilard et al., 2021).

Proximity to their homes, easy reaching of different places, availability of appropriate benches, absence of architectural barriers, a good quality of lighting and the absence of *incivilities*, are some of the key features of a public space tailored to older citizens. The latter two are particularly important in defining the feeling of security experienced in the public space. For older people, as for other groups of citizens considered as socially frail and vulnerable, the feeling of safety is an essential condition for a full and a comprehensive experience of the city, in terms of both time and space. The “geography of fear” (Carrera, 2015) makes the city smaller, denying in fact a large number of people the opportunity to travel across and fully enjoy the city. This means that the affirmation of “the right to the city” is, thus, undermined. Building the conditions of a safe public space, firstly by reinforcing the sense of security perceived by the citizens, is therefore a fundamental action to contrast the *peripheralization* of cities and of some areas of them and the widespread sense of insecurity that keeps seniors citizens, more than others, away from urban public spaces and turns the city into an archipelago of fragmented spaces (Magatti, 2009, 2018; Carrera, 2015; Petrillo, 2018). Designing public space properly and functionally means ensuring one of the conditions for sociability and counteracting relational poverty.

However, this opens up a further reflection around the characteristics and the potentiality of public space. The availability of public spaces, such as streets and squares, does not fully guarantee that the conditions for sociality are met. If encounters occur within these places, they are fleeting and ephemeral. This kind of public space – that could be defined as “*passageways*” (Carrera, 2022a) – does not offer the right conditions for mutual acquaintance and recognition, or for the creation of bonds. Compared to other groups of citizens, the older people need for public “*relational spaces*” where they can stay, encounter and build relationships.

This shows how crucial urban planning can be in creating the conditions for the use of public spaces as well as in connecting and coordinating citizens’ different needs. For older people it can be translated in the promotion of their right to an active aging and in their “active engagement” (Sandercook, 2003).

### The potentiality of “third space”

The notion of third spaces can help to overcome the limits of the public space as “passageways” as defined before. The third space does not solve the antimony between *agorà* and *oikos* (Cacciari, 2004), but offers a *third* option because of its distinctive physical, practical, symbolic and cultural features. Third space refers to a set of public and semi-public spaces, a plurality of small, interstitial places scattered and widespread trough the city whose specific characteristics may offer the opportunity to implement strategies of inclusion and encounter. It concerns gardens, schools, universities, community libraries, sports clubs, leisure centres and other public spaces within the neighborhoods (like, for example, the “community houses” in Italy), or places upgraded with the same purpose of providing *public micro-spaces* for social encounters (Carrera, 2021). These urban spaces can assume a particularly intense meaning and even nurture a shared sense of belonging (Ambrosini, 2013). Thinking of such places, above all others, it is possible to understand what Susan Halford (2004) meant when she wrote that “the sense of space develops in line with its use.”

As Bhabha (2001) and Edward Soja (1996, 2007) suggest in their analysis, the *third space* can be conceived of as a feature of post-modernity culture that emphasizes the role of the space and its symbolic representation and function. It points to a horizon of new, somehow liminal, interstitial spaces that allow the social construction and the construction of radical changes, enabling creative responses to changes occurring within the urban space. The *third space* creates opportunities for relationships, cultural and leisure initiatives between different people, who may establish more long-term forms of associations (Carrera, 2022a,b).

In the process of creating the conditions for a different model of relationality, the *third space* is a key approach. The aim is to create a urban micro-spaces network that can help to ease the weight of age differences – but not only concerning them –through the everyday life routines, by establishing common interests and encouraging people to share new life projects within this specific kind of space, new form of interest communities can take place (Settersen and Angel, 2011; Greenfield et al., 2019; Manzini, 2021).

As Alfredo Mela writes, “urban practices rely on what we might call the infrastructural endowments of each city” (Mela, 2020, p. 49). Based on this assumption, it is possible to argue that acting on infrastructures can be a way to influence the practices. Without falling into a deterministic perspective, it is possible to see daily practices as forms of active adaptation to the urban environment. Thus, space is a factor that can contribute to define not only the opportunities and limits for action and *capabilities* (Sen, 1986, 1992; Nussbaum, 2002), but also the meaning that these assume (Mela, 2020, p. 91). In relation to this, it is worth to recall the concept of “affordance” (Gibson, 1979), according to which, by offering potential opportunities, the space determines the framework within which different subjects can act and make their choices.

Therefore, re-designing a high-quality urban space targeted and disseminated by structural transformations is a key strategy for the implementation of full inclusion processes. When included in a medium and long-term planning, these *urban acupunctures* aim to counteract and overcome both explicit and implicit forms of social exclusion and relational poverty. These small-scale interventions can be the outcome of bottom-up processes or co-planning activities among experts, administrators, citizens and other urban stakeholders, enhance local social and cultural resources. They generate virtuous circles of sustainable, participatory and inclusive urban and social development and can help reinforcing or building up again the sense of community and belonging.

Within this process, the neighborhoods, more than the city as whole, can be real laboratories for reconstructing widespread systems of mutual care and relationships. As Jaime Lerner argues (2003), despite the small scale of the interventions in terms of architectural-urban planning and, sometimes, also in terms of costs, these urban and social acupunctures can generate a great potential for change, a domino effect that can also cover very large areas and deeply affect the quality of life (Lerner, 2003). In this context, third spaces can help to deal with the challenges related with the aging process. They can create the material conditions for an effective transformation of the city into an age-friendly place and, moreover, for create an age-friendly communities enhancing the emotional life-space level (Greenfield et al., 2015; Moulaert and Garon, 2016; Scharlach and Lehning, 2016; Buffel and Phillipson, 2018).

### The private space

As said, private space plays also a key role in ensuring the conditions for a high life quality. If the environmental urban context is recognized as a decisive element in ensuring full rights of aging in place, a safe and accessible home environment is not less important and decisive. The material changes to guarantee a better use of spaces, and changes related to the intangible conditions of living are very heavy conditions of older people lives. If the first concerns the removal of certain architectural barriers, and hence depends above all on personal decisions, the second concerns the need to rethink the idea of domestic space in view of the purpose of improving sociality preventing relational poverty.

Some effective tools to avoid older people the experience of social isolation and feeling of loneliness are the co-housing, the senior housing and the new forms of housing inspired by the *beguinage* (in Dutch “Begijnhof”). The first model provides opportunities for cohabitation, between seniors people and young couples, while the second offers a possible solution to both the difficulty of finding a house, − which has been significantly accentuated by the ongoing economic crisis -, and the need for childcare, which is an historical deficiency of the Italian social welfare system. This latter model, born in Northern Europe, precisely in Denmark at the turn of the Seventies, has also been experimented in Italy in the last few years. It is not just a new housing model, but a real new lifestyle that ensures an active existential dimension and represents a worthy possibility to preserve adequate levels of autonomy and quality of life. At the heart of this new different form of living there are some shared spaces within private houses, where the seniors can experience new patterns of encounter and relationships (Kesserling et al., 2015). Sometimes these solutions can be offered to older people with disadvantaged social-economic backgrounds. The third model, however, refers to an independent community of Beguines, typical of Northern Europe, consisting of a group of integrated buildings, built usually around a tree-lined courtyard, which includes not only domestic and monastic structures, but also the laboratories used by the community and an infirmary.

Despite strong architectural and functional differences, these models of rethinking home living have in common *red thread* of specific objectives, that can be listed as following: (a) de-institutionalization and socialization in favor of older citizens, with a view on integrating private and public provision of services, aimed at promoting the quality of life and ensuring the right to a full quality process of aging; (b) ability to recognize the risk of social isolation and to counteract them through different lifestyles implying experiences of sharing and daily cohabitation; (c) reinforcing the idea of older people with skills and resources useful self and mutual care; (d) overcoming situations of loneliness, sometimes abandonment, and socio-economic hardship through the reception of these model of shared home; (e) strengthening the quantity and quality of links with the informal network (family, friends, volunteers, etc.); (f) involvement of the older people in decision-making and practical processes; greater self-care, in terms of physical and psychological health; and active role in supporting the social plan shared with the Territorial Social Services (Carrera, 2022b, p. 8).

The complex nature of these objectives explains the need for coordinated interdisciplinary and multifactorial actions in which public welfare, social cooperatives and housing associations work together to enable well-structured territorial networks to create a functional urban habitat.

## Conclusion

Urban space policies are important tools to address the complexity of the aging process and to deal with its challenge. Space, or rather urban spaces, are not just neutral scenarios of the citizens’ practices but essential conditions that can facilitate or hinder those practices. It is necessary to re-conceptualize the quality of life in terms of an interrelation between urban spaces or, more in general, urban habitat, and the principles of social justice and territorial democracy (Carrera, 2020), starting with the transformation of social representations and self-representations of seniors people. Both the principles underpinning the Lefebvrian “right to the city” for every citizen, acquire a special significance for older subjects. Cities that are organized around the use of vehicles, with undersized and often technically unsuitable public transport, long streets for the shopping, not well-equipped squares, narrow and bad maintained pavements that increase the risk of falls, the lack of attention to pedestrianism and pedestrianization, the scarcity of social, recreational and cultural facilities and services often concentrated only in a few areas of the city, especially the central ones, places lacking a diversified offer and not always free, all these mentioned are not “places for all,” and surely not suitable for the older citizens and city users (Iwarsson et al., 2007).

The right to an urban high-quality habitat is strictly linked with an adequate level of differentiated functional and relational proximity and social connections (Evans et al., 2002; Iwarsson et al., 2007; Herbers and Mulder, 2017). “Inclusive or excluding social processes find an effective materialization in the way the city and its public spaces are built, in the distribution of urban space, in the characteristics of services location and public places” (Ciaffi et al., 2020, p. 66). And, as Secchi writes: “the main features of the city and the territorial project are, at the same time, the main virtuous or perverse devices of well-being” (Secchi, 2013, p. 24). The increasingly differentiated and pressing demands of older citizens are focused on accessible public spaces, territorial medicine centers, home services, proximity, mobility, evenly distributed throughout the urban territory. In view of achieving the goal of an inclusive and age-friendly city, the strategies just outlined should become a priority for public policy makers. The need to create, activate and coordinate a wide network of different territorial subjects becomes central political issue to pursue the goal of projecting a material and social urban habitat adequate for older people’s needs and desires.

This analysis opens to later studies to analyze how urban policies could be able to support the age-friendly cities and, even more than this, to enhance the conditions for age-friendly communities.

## Data availability statement

The original contributions presented in the study are included in the article/supplementary material, further inquiries can be directed to the corresponding author.

## Author contributions

LC: Conceptualization, Writing – original draft.

## Footnotes

^1^Emily Greenfield, Kathy Black and Tine Buffel observe that very fa from a shift in ageing paradigm: “few public resources have gone toward supporting independent living in the community because the medical profession, business and government are each more comfortable with a skilled nursing institutional model of long-term care that serves as extension of acute care medicine, which allows for ready profit making, and limits social expenditures” (Greenfield et al., 2019, p. 4).

^2^“Social isolation can be defined as an objective measure reflecting an individual’s lack of contact or ties with others, such as family, friends, acquaintances and neighbors. Social isolation is characterized as an absence or limitation in the quantity of social interactions. It is distinct from the related and subjective concept of loneliness which occurs when there is a perceived discrepancy between an individual’s desired and achieved level of social interaction” (de Jong-Gierveld et al., 2006).
